# Association between Metabolic Syndrome and Cognitive Impairment after Acute Ischemic Stroke: A Cross-Sectional Study in a Chinese Population

**DOI:** 10.1371/journal.pone.0167327

**Published:** 2016-12-09

**Authors:** Pan Li, Wei Quan, Da Lu, Yan Wang, Hui-Hong Zhang, Shuai Liu, Rong-Cai Jiang, Yu-Ying Zhou

**Affiliations:** 1 Department of Neurology, Tianjin Huanhu Hospital, Tianjin, P. R. China; 2 Tianjin Key Laboratory of Cerebral Vascular and Neurodegenerative Diseases, Tianjin Neurosurgery Institute, Tianjin Huanhu Hospital, Tianjin, P. R. China; 3 Department of Neurosurgery, Tianjin Medical University, General Hospital, Tianjin, P.R. China; 4 Tianjin Neurological Institute, Key Laboratory of Post-trauma Neuro-repair and Regeneration in Central Nervous System, Ministry of Education, General Hospital, Tianjin, P.R. China; 5 Tianjin Key Laboratory of Injuries, Variations and Regeneration of Nervous System, General Hospital, Tianjin, P.R. China; Banner Alzheimer's Institute, UNITED STATES

## Abstract

**Background and Objectives:**

Metabolic syndrome (MetS), a risk factor for many vascular conditions, is associated with vascular cognitive disorders. The objective of the present study was to explore the associations of MetS and its individual components with the risks of cognitive impairment and neurological dysfunction in patients after acute stroke.

**Methods:**

This cross-sectional study enrolled 840 patients ranging in age from 53 to 89 years from the Tianjin area of North China. Cognitive function was evaluated using the Montreal Cognitive Assessment (MoCA) and Mini-Mental State Examination. Neuropsychiatric behavior was assessed using the Neuropsychiatric Inventory Questionnaire. Emotional state was examined according to the Hamilton Depression Rating Scale, and neuromotor function was evaluated using the National Institutes of Health Stroke Scale, Barthel index, and the Activity of Daily Living test. After overnight fasting, blood samples were obtained to measure biochemistry indicators.

**Results:**

MetS and its individual components were closely correlated with MoCA score. MetS patients had high levels of inflammation and a 3.542-fold increased odds ratio (OR) for cognitive impairment [95% confidence interval (CI): 1.972–6.361]. Of the individual MetS components, central obesity (OR 3.039; 95% CI: 1.839–5.023), high fasting plasma glucose (OR 1.915; 95% CI: 1.016–3.607), and type 2 diabetes (OR 2.241; 95% CI: 1.630–3.081) were associated with an increased incidence of cognitive impairment. Consistent and significant worsening in different neurological domains was observed with greater numbers of MetS components.

**Conclusions:**

MetS was associated with worse cognitive function, neuromotor dysfunction, and neuropsychological symptoms among Chinese acute stroke patients.

## Introduction

Metabolic syndrome (MetS) is the name given to a cluster of metabolic abnormalities, including obesity (particularly central adiposity), hyperglycemia, hypertension, hypertriglyceridemia, and decreased high density lipoprotein cholesterol (HDL-C) [1]. Most of these conditions have been shown to be independent risk factors for stroke. MetS itself was already shown to increase the risks of both clinical stroke and silent brain infarction by 2–4 times [2, 3]. Moreover, the role of MetS in the development of vascular disease has led to the discovery of its influence on cognitive decline, overall dementia, and vascular dementia (VaD) in particular [4].

In recent years, the associations between MetS and the risks of Alzheimer disease (AD) and other forms of cognitive impairment have been studied extensively. However, the effect of MetS on the rate of cognitive decline remains controversial [5–12]. Multiple studies have reported increases of 2–7-fold in the risk of developing cognitive impairment among MetS patients [7, 8]. Other studies found that the occurrence of MetS is associated with an increased risk of incident VaD but not of AD or other dementias during follow-up [9, 10], but still yet other studies failed to find such an increased risk [11, 12]. These conflicting results can be attributed to differences across gender, age, and ethnic groups in these previous studies. In China, with respect to the high prevalence rate of MetS, even a modest association with cognitive impairment could have considerable implications for public health. Early identification and treatment of individuals with MetS among stroke patients could help to alleviate or prevent the development of cognitive impairment in these patients.

In the present study, we examined the association between MetS and the risk of developing cognitive impairment in a Chinese population of acute ischemic stroke patients. We additionally assessed such relationships for individual components of MetS and whether each unit increase in the number of MetS components further increases the risk of cognitive impairment after acute ischemic stroke.

## Materials and Methods

### Patient selection and exclusion criteria

This cross-sectional study was conducted through a collaboration of the Cerebral Vascular and Neurodegenerative Disease Center and the Neurology Department of Tianjin Huanhu Hospital in Tianjin, China. Eight hundred forty consecutive registered inpatients treated for acute ischemic stroke (within 7 days of onset) between December 2010 and October 2015 were enrolled according to the current consensus diagnostic criteria [13]. Participants were included only if the diagnosis of acute ischemic stroke was confirmed on brain magnetic resonance imaging (MRI) or computed tomography (CT) scanning. The exclusion criteria were: 1) aphasia, severe illnesses, and difficulty completing the procedures of the study; 2) a pattern of deficits better explained by another neurological disease, cerebral trauma, tumor, intoxication, or metabolic or inflammatory disorders; 3) serious chronic conditions within the previous year; 4) biomarker findings that strongly indicated AD or other forms of dementia; 5) a history of psychiatric disorders or abnormal behavior; and 6) the existence of cognitive impairment before onset of the present stroke. The diagnosis of MetS was determined according to criteria proposed by the National Cholesterol Education Program (NCEP) Adult Treatment Panel III (ATP III) [1], the International Diabetes Federation [14], and the American Heart Association [15]. These guidelines suggest the MetS exists if a person has three or more of the following criteria: 1) central obesity [waist circumference >102/90 cm for men or >88/80 cm for women or body mass index (BMI) ≥30 kg/m^2^ based on the criterion used by the World Health Organization]; 2) elevated plasma triglycerides (TG) (≥150 mg/dl; 1.695 mmol/l); 3) low HDL-C (<40 mg/dl, 1.036 mmol/l for men or <50 mg/dl, 1.295mmol/l for women); 4) high blood pressure (≥130/85 mmHg) or receiving hypertensive treatment; and 5) high fasting plasma glucose (FPG) (≥110 mg/dl, 6.105 mmol/l).

### Ethical considerations

Written informed consent was obtained from all participants and their assigned surrogate decision-makers (spouse or child). A reliable caregiver accompanied the patient at all study visits and supervised their treatment. If a patient’s cognitive disability made it impossible for them to provide valid informed consent, we acquired written informed consent from the patient’s surrogate (spouse or child). All participants were free to withdraw from the study at any time and without giving a reason. This study was approved by the local institutional ethical standards committee on human experimentation of Tianjin Huanhu Hospital (Tianjin, China) and was conducted in accordance with the Declaration of Helsinki.

### General data collection

General patient information, including age, gender, ethnicity, educational level, current smoking status, alcohol use, marital status, and family history of dementia, was collected at study onset. Prior diagnoses of diabetes, hypertension, heart disease, cerebrovascular disease, accompanying endocrine disorders, brain trauma, brain tumor, and hematopathy as well as any history of poisoning were identified in the first interview with participants. Participants were asked to show all their current medications and report the details of their drug use. Height and weight were measured while participants wore indoor clothing and no shoes, and BMI was calculated as the weight in kilograms divided by the height in meters squared. Waist circumference was measured between the lower rib margin and the iliac crest after a normal expiration. Arterial blood pressure was measured twice with the participant in a seated position using a digital electronic tensiometer, and the average value was used in the statistical analysis.

### Assessment of biochemical indices

Morning blood or serum samples were collected after an overnight fast on the next day after admission to the hospital. FPG, total cholesterol (TC), TG, HDL-C, and low-density lipoprotein cholesterol (LDL-C) levels were measured with the ADVIA 2400 full automatic biochemical analyzer (Siemens, Germany). Serum hypersensitive-C reaction protein (hs-CRP), glycosylated hemoglobin (HbA1c), and homocysteine (Hcy) levels were detected by the BNP II protein analyzer (Siemens).

### Evaluation of neurological function

Five general areas of neurological functions, including subscale scores, were evaluated: cognitive function using the Chinese versions of the Montreal Cognitive Assessment (MoCA) and Mini-Mental State Examination (MMSE), neuropsychiatric behavior using the Neuropsychiatric Inventory Questionnaire (NPI-Q), emotional state using the Hamilton Depression Rating Scale (HAMD), activities of daily living using the Activities of Daily Living (ADL) test, and neurological function using the National Institutes of Health Stroke Scale (NIHSS) and Barthel index (BI). The MoCA [16] and MMSE [17] scores both have ranges from 0 to 30, with higher scores indicating better cognitive function. The NPI-Q is used to measure the frequency and severity of behavioral disturbances in patients with dementia [18]. The HADM is a standard instrument used for determining emotional state [19]. The ADL scale was developed specifically to evaluate the functional abilities of patients with cognitive impairment [20]. The NIHSS has been widely used to evaluate the severity of symptoms and signs in patients with stroke (0 points indicates normal neurological function) [21]. For the NPI-Q, HADM, ADL, and NIHSS, higher scores indicate greater impairment. The BI was applied to measure the living ability of patients with neurologic deficits. This index can be divided into five ranks according to the dependence of the patient on others for help, with higher scores indicating better performance [22]. In addition, the Hachinski Ischemic Score (HIS) is the favored clinical tool used for diagnosis of vascular dementia. A score greater than 7 suggests vascular involvement [23]. Data for all participants were reviewed by an expert panel of neurologists, neuropsychologists, and nurses. The diagnostic procedures consisted of medical history documentation, psychiatric and neurological examination, and laboratory screening. Final diagnosis in every case required consensus among at least two experienced neurologists.

### Statistical analysis

Baseline demographic qualitative variables were analyzed with the Chi-square test and described as the relative abundance ratio (%) or rate (%). Normally distributed quantitative variables were analyzed with two independent sample t-tests or one-way analysis of variance (ANOVA) and are reported as mean±standard deviation (SD). The relationships between MoCA score and other clinical characteristics were assessed using partial correlation coefficients. Binomial or multinominal logistic regressions were performed to explore the associations of MetS, the number of MetS components, and individual components of MetS with cognitive function and other variables. The strength of each association was evaluated according to the odds ratio (OR) and corresponding 95% confidence interval (CI). The age, gender, education level, marital status, smoking status, alcohol drinking, stroke history, family history of dementia, neuropsychological scores, and medication status of each participant were imported as covariates in the statistical analysis. All statistical analyses were performed using SPSS software, version 21.0 (SPSS, Inc., USA). All tests were two-tailed, and *P* values <0.05 were considered statistically significant.

## Results

Among the 840 patients included in this study, the mean age was 62.31±11.15 years (range, 53–89 years), and 41.1% of study participants were female. A diagnosis of MetS was made in 330 (39.3%) cases. Thirty-seven (4.4%) patients presented none of the five components of MetS, whereas 184 (21.9%) and 289 (34.4%) patients presented 1 or 2 components of MetS, respectively.

Table 1 displays the baseline characteristics of the patient groups with and without MetS. Patients with MetS exhibited a higher prevalence of hypertension, type 2 diabetes (T2D), and stroke history as well as a larger mean waist circumference, higher BMI, higher systolic and diastolic blood pressure, higher levels of TC, LDL-C, Hcy, hs-CRP, TG, FPG, and HbA1c, and a lower level of HDL-C than stroke patients without MetS. In addition, current smokers and drinkers were more likely to have MetS than those who did not smoke or drink alcohol. Furthermore, stroke patients with the MetS showed a greater prevalence of cognitive impairment (251 cases, 29.9%) than those without MetS (204 cases, 24.3%; *P*<0.001). No significant differences were observed for the other variables between the patient groups with and without MetS.

**Table 1:** Baseline demographics and clinical characteristics of Non-MetS and MetS groups.

Variables	Non-MetS	MetS	Statistic value	*P* value
**Enrolled patients (n [%])**	510 (60.71)	330 (39.29)	—	—
**Age (mean±SD, yrs.)**	62.29±10.49	62.30±12.11	-0.016^b^	0.987
**Gender (M/F)**	309/201	186/144	1.477^a^	0.224
**Education (Mean±SD, yrs.)**	8.86±2.08	9.23±2.14	-1.292^b^	0.197
**CHD (n [%])**	55 (10.78)	27 (8.18)	1.540^a^	0.215
**Smoking (n [%])**	280 (54.90)	206 (62.42)	4.650^a^	0.031*
**Drinking (n [%])**	194 (38.04)	153 (46.36)	5.727^a^	0.017*
**Stroke history (n [%])**	252 (49.41)	221 (66.97)	25.106^a^	0.000*
**Dementia family history (n [%])**	20 (3.92)	19 (5.76)	1.526^a^	0.217
**Waist circumference (Mean±SD, cm)**	82.34±4. 89	97.88±7.39	-2.741^b^	0.006*
**BMI (Mean±SD, kg/m**^**2**^**)**	24.74±2.89	26.17±4.03	-5.950^b^	0.000*
**BMI ≥ 30 kg/m**^**2**^ **(n [%])**	25 (4.90)	39 (11.81)	13.616^a^	0.000*
**TC (Mean±SD, mmol/l)**	4.87±1.16	5.10±1.06	-2.864^b^	0.004*
**LDL-C (Mean±SD, mmol/l)**	2.90±0.92	3.07±0.86	-2.697^b^	0.007*
**Hcy (Mean±SD, mmol/l)**	13.36±2.71	15.84±3.99	-3.303 ^b^	0.001*
**hs-CRP (Mean±SD, mg/l)**	2.07±0.50	3.88±0.66	-8.552 ^b^	0.000*
**TG (Mean±SD, mmol/l)**	1.57±0.11	1.72±0.37	-1.999^b^	0.046*
**TG ≥1.695 mmol/l (n [%])**	82 (16.08)	246 (74.55)	287.767^a^	0.000*
**HDL-C (Mean±SD, mmol/l)**	1.17±0.41	1.01±0.26	2.697^b^	0.007*
**HDL-C < 1.036 (M); < 1.295 (F) mmol/l (n [%])**	317 (62.16)	315 (95.45)	119.237^a^	0.000*
**SBP (Mean±SD, mmHg)**	142.59±15.36	158.42±19.15	-2.795 ^b^	0.005*
**DBP (Mean±SD, mmHg)**	83.61±14.40	91.33±16.97	-2.416 ^b^	0.016*
**Hypertension (n [%])**	261 (51.18)	206 (62.42)	10.268^a^	0.001*
**FPG (Mean±SD, mmol/l)**	5.85±1.04	6.44±1.67	-3.766^b^	0.000*
**FPG ≥ 6.105 mmol/l (n [%])**	70 (13.73)	213 (64.55)	231.628^a^	0.000*
**HbA1c (Mean±SD, %)**	6.12±0.90	6.95±1.49	-5.622^b^	0.000*
**T2D (n [%])**	207 (40.59)	218 (66.06)	52.007^a^	0.000*
**cognitive normal (n [%])**	306 (36.43)	79 (9.40)	104.944^a^	0.000*
**cognitive impairment (n [%])**	204 (24.29)	251 (29.88)		

^a^ is *χ*^*2*^ statistic value and analyzed with Chi-square test,

^b^ is *t* statistic value and analyzed with independent sample t-tests;

* *P*<0.05 vs. Non-MetS group.

Non-MetS: non-Metabolic syndrome; MetS: Metabolic syndrome; M: male; F: female; CHD: Coronary Heart Disease; BMI: body mass index; TC: total cholesterol; LDL-C: low-density lipoprotein cholesterol; Hcy homocysteine; hs-CRP: high-sensitivity C-reactive protein; TG: triglyceride; HDL-C: high-density lipoprotein cholesterol; SBP: systolic blood pressure; DBP: diastolic blood pressure; FPG: Fasting plasma glucose; HbA1c: glycosylated hemoglobin; T2D: type 2 diabetes.

Table 2 presents the results of our analysis of different neurological functional domains. The mean MMSE and MoCA scores were much lower for stroke patients with MetS than for stroke patients without MetS (MMSE: 24.27±5.12 *vs* 22.03±3.06, *P* = 0.031; MoCA: 21.32±6.41 *vs* 18.05±4.67, *P* = 0.005). Moreover, the MetS group had higher scores on the NPI-Q, HAMD, and HIS, but a lower BI compared with the group of patients without MetS, indicating the greater occurrence of cognitive and functional deterioration in stroke patients with MetS. The ADL score did not differ significantly between the two patient groups.

**Table 2:** Comparison of neuropsychological scale scores between Non-MetS and MetS groups (n = 840).

Variables	Non-MetS	MetS	*t* value	*P* value
**MMSE**	24.27±5.12	22.03±3.06	2.157	0.031*
**MoCA**	21.32±6.41	18.05±4.67	2.810	0.005*
**NIHSS**	3.20±0.73	3.77±1.05	-2.616	0.009*
**BI**	85.92±12.27	83.24±14.96	2.492	0.013*
**ADL**	26.48±5.10	27.45±4.24	-1.041	0.298
**NPI-Q**	2.72±0.54	3.68±0.79	-2.181	0.030*
**HAMD**	4.34±0.54	5.19±1.14	-3.870	0.000*
**HIS**	7.12±1.68	8.90±1.41	-7.287	0.000*

Data are mean±SD; analyzed with independent sample t-tests, MMSE: Mini-Mental State Examination; MoCA: Montreal Cognitive Assessment; NIHSS, National Institutes of Health Stroke Scale; BI: Barthel Index; ADL: Activity of Daily Life; NPI-Q: Neuropsychiatric Inventory Questionnaire; HAMD: Hamilton Depression Rating Scale; HIS: Hachinski ischemic scale.

* *P* < 0.05 vs. Non-MetS group.

The statistical values for the correlation of cognitive level and the clinical characteristics of study participants are listed in Table 3. The MoCA score was negatively correlated with BMI, TG, SBP, DBP, FPG, and the number of MetS risk factors, but positively correlated with the HDL-C level in all study participants.

**Table 3:** Correlation of MoCA Score and MetS components in the study participants (n = 840).

Variables	*R* value	*P* value*
**BMI**	-0.723	**0.000**
**TG**	-0.302	**0.000**
**HDL-C**	0.694	**0.000**
**SBP**	-0.726	**0.000**
**DBP**	-0.308	**0.000**
**FPG**	-0.277	**0.000**
**Number of MetS components**	-0.579	**0.000**

* Partial correlation analysis, *P*<0.05; adjusted for age, gender, education, marriage status, smoking status, alcohol drinking, stroke history, family history of dementia, neuropsychological scores and medication status (aspirin, statins, diabetes drugs, regulating blood pressure drugs, neurotrophic drugs, and others), etc.

The risk factors for cognitive impairment identified by multivariate logistical regression in all participants are listed in Table 4. In the unadjusted model, MetS was significantly associated with increased odds of cognitive impairment (OR 2.707; 95% CI: 1.763–4.155). Adjustment for sociodemographic and clinical covariates led to an increase in the estimated OR value (OR 3.542; 95% CI: 1.972–6.361). For individual MetS components, patients with a larger BMI, elevated FPG level, and history of T2D had a significantly higher risk of having cognitive impairment than did patients without these health problems in both the unadjusted and adjusted models. Furthermore, an increasing number of MetS components was significantly associated with cognitive impairment in both models.

**Table 4:** Associations of MetS and its components with cognitive impairment in all participants (n = 840).

Variables	Model 1		Model 2	
	OR (95% CI)	*P*	OR (95% CI)	*P*
**MetS**	2.707 (1.763–4.155)	0.000*	3.542 (1.972–6.361)	0.000*
**BMI**	1.099 (1.032–1.171)	0.003*	3.039 (1.839–5.023)	0.000*
**TG**	1.054 (0.906–1.227)	0.497	1.124 (0.937–1.350)	0.208
**HDL-C**	0.673 (0.342–1.324)	0.251	0.654 (0.286–1.495)	0.314
**Hypertension**	1.064 (0.972–1.164)	0.181	1.054 (0.992–1.120)	0.091
**SBP**	1.000 (0.667–1.499)	0.998	0.727 (0.448–1.178)	0.195
**DBP**	1.078 (0.968–1.201)	0.171	0.985 (0.963–1.006)	0.164
**FPG**	1.138 (1.029–1.258)	0.012*	1.915 (1.016–3.607)	0.044*
**T2D**	1.133 (1.007–1.275)	0.038*	2.241 (1.630–3.081)	0.000*
**Number of MetS components**	1.314 (1.059–1.630)	0.013*	1.431 (1.101–1.860)	0.007*

Model 1: unadjusted model; Model 2: adjusted for age, gender, education, marriage status, smoking, alcohol drinking, stroke history, family history of dementia, neuropsychological scores and medication status (aspirin, statins, diabetes drugs, regulating blood pressure drugs, neurotrophic drugs, and others), etc. OR: odds ratio; CI: confidence interval.

* binary logistical regression analysis, *P*<0.05.

**Fig 1:**
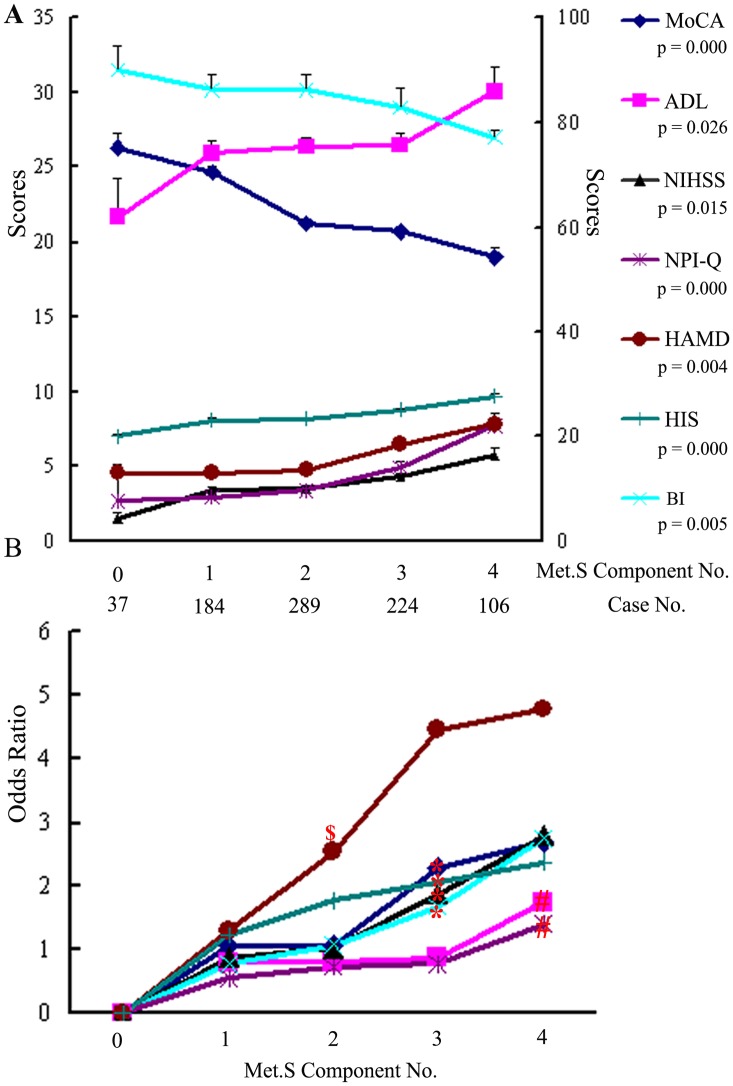
Stratified analysis of association between number of MetS risk factors and different neurological domains. (A) Test variable scores according to the number of MetS components. Data are mean±SD; ANOVA analysis. (B) OR for lower performance in the different neurofunctional tests according to the number of MetS components. Multinominal logistical regression for all values, $ indicates *P*<0.05 when the number of MetS components was two; * indicates *P*<0.05 when the number of MetS components was three; # indicates *P*<0.05 when the number of MetS components was four or more.

Fig 1A depicts the cognitive (MoCA), neuromotor (NIHSS), physical function (ADL and BI), neuropsychiatric behavior (NPI-Q), affective (HADM), and ischemic (HIS) variable scores according to the number of MetS risk factors. The results revealed a consistent and significant worsening of these scores with an increasing number of MetS components (*P*<0.05 for all). Fig 1B shows the respective ORs for the investigated neurofunctional variables. All variables were associated with a significantly higher risk for lower performance as the number of MetS components increased. Participates with two or more risk components had a more than 2-fold higher chance of impairment on the HAMD (red $ indicates statistical significance). When the number of risk factors reached three or more, participants exhibited worsening performance on the MoCA, NIHSS, BI, and HIS scores (red * indicates statistical significance). However, participant showed deteriorated performance on the NPI-Q and ADL only when the number of risk factors reached 4 or greater (red # indicates statistical significance).

## Discussion

Our study demonstrated correlations between cognitive impairment and MetS in a group of acute cerebral ischemia patients in China. In addition to the overall association, for every unit increase in the number of MetS components, the risk of accelerated cognitive impairment and neurological dysfunction also increased. Among the five components of MetS, abdominal obesity, a high FPG level, and history of T2D were most common among patients who developed cognitive impairment even after adjusting for associated confounding factors. Therefore, clinicians should be aware of this clinical association, as early identification and treatment of these risk factors could offer new avenues for disease course modification.

MetS is characterized by a clustering of risk factors for vascular disease, and in recent studies, MetS has been shown to be an important potential contributor to worsening cognitive impairment [4–12]. Our findings add to a growing body of evidence that suggests MetS is associated with accelerated cognitive aging and the risk of cognitive impairment in acute cerebral ischemia patients, independently of other known risk factors for cognitive impairment (OR 3.542; 95% CI:1.972–6.361, Table 4). This correlation is consistent with the findings of other studies in Chinese populations [5, 6, 24]. However, the overall prevalence of MetS in the present study was 39.3%, which differed from values reported for other Chinese population-based studies [5, 6, 24]. This discrepancy may be explained by differences in the characteristics of the study populations. In addition, we found that with an increase from zero to three or more MetS components, performance on the cognition, neuromotor, physical function, neuropsychiatric behavior, and affective scales worsened significantly (Fig 1A). Accordingly, there was a consistent and significantly higher risk for impairment in these respective domains with an increasing number of MetS components. For all functional scales except the HAMD, the cut-off point was equal to or greater than three MetS components (Fig 1B). Notably, four major cross-sectional and longitudinal studies have confirmed the proposed association between MetS and VaD [9, 10, 25, 26]. The Honolulu Asia Aging Study (HAAS) was the first prospective cohort study of cardiovascular disease that was extended 25 years later to investigate associations with dementia and revealed an increased risk of dementia with the clustering of individual MetS components. In the HAAS, a clustering of seven metabolic vascular risk factors (random postload glucose, SBP, DBP, BMI, subscapular skinfold thickness, TG, and TC) increased the risk of dementia, and in particular the risk of VaD, but not AD [25]. Raffaitin et al. and Solfrizzi et al reported the only two longitudinal population-based studies that lasted more than 3 years and evaluated whether MetS is a risk factor for VaD [9, 10]. Both studies indicated that a MetS diagnosis at baseline was associated with an increased risk of incident VaD. Moreover, MetS patients with a high inflammation status showed 9.5-fold increased risk of VaD compared with patients without MetS [10]. These studies also found that a high TG level, hypertension, and type 2 diabetes were significantly associated with a higher incidence of VaD among the individual components of MetS [9, 10]. Not all studies, however, have reported a significant association between MetS and cognitive decline [11, 12]. Muller and colleagues found no significant association between MetS and prevalent dementia, but found that the individual components of type 2 diabetes and hyperinsulinemia were associated with an increased incidence of AD [12].

In the present study, we found that among the individual MetS components, the principal components of central obesity, high FPG level, and medical history of T2D significantly influenced the prevalence of cognitive impairment among the study population. Notably, the worldwide prevalence of obesity has more than doubled since 1980, with approximately 39% of adults being overweight in 2014 and approximately 13% being obese (http://www.who.int/mediacentre/factsheets/fs311/en/). The incidence rate of central obesity in our study population was 7.6%, and high BMI values corresponding to a 3.039-fold increased OR for cognitive impairment after controlling for potential confounding factors. A previous study demonstrated that the association between BMI quartiles and the risk of dementia resembles a “U” shaped-curve [27]. Both too high (>29 kg/m^2^) or too low (<21 kg/m^2^) BMI values have been associated with an increased risk of cognitive decline [28], possibly due to the poor nutrition related to lower BMI levels and the increased risk of vascular conditions at higher BMI levels. Furthermore, the influence of obesity on cognitive impairment is also closely related to the age of the research cohort, and this age-dependence likely explains the paradoxical relationship between obesity and dementia across studies [29]. Diabetes, specifically T2D, along with a higher FPG are highly prevalent conditions among older adults. In addition, the combination of obesity and T2D leads to diminished performance across various cognitive domains [30]. Obesity induces hormonal abnormalities and peripheral insulin resistance (IR), which can alter central insulin signaling and lead to AD-like cognitive impairment [31, 32]. In our study, the separate impacts of T2D and an elevated FPG on cognitive function after stroke were statistically significant, supporting the notion that T2D and impaired fasting glucose are risk factors for cognitive impairment.

The mechanisms underlying the association between MetS and cognitive impairment after acute cerebral ischemia remain incompletely understood. Several hypotheses have been proposed to explain this relationship. One of the simplest plausible pathways is that both MetS and cognitive function are affected by cerebrovascular disease [4, 33]. Macrovascular and microvascular injury caused by diabetes or hyperglycemia can in turn contribute to the development of both AD and vascular cognitive disorders [4, 33, 34]. Another probable mechanism is based on the effects of the elevated inflammation and reactivated oxidative stress reaction often seen in the setting of MetS [35]. Both can produce direct effects on the pathogenesis of neurofibrillary tangles and β-amyloid protein (Aβ) formation [36]. A previous study found that participants with MetS and high levels of inflammation had a 2.3-fold greater OR for non-amnestic mild cognitive impairment [37]. In our investigation, participants with MetS had higher concentrations of hs-CRP and Hcy, both of which have been demonstrated to be associated with increased risks of developing dementia and cognitive decline [37, 38]. In addition, IR, a well-established contributor of the primary pathophysiology in MetS, is strongly implicated in the etiopathology of dementia [31]. Peripheral MetS induces central IR in the brain. The resulting impaired insulin signaling, which mainly impacts the PI3K/Akt pathway, then increases amyloid precursor protein (APP) processing/Aβ levels and tau phosphorylation. Increased Aβ production further disrupts insulin signaling to exacerbate the AD pathology and cognitive decline [31]. Finally, we not only found that MetS was individually associated with lower cognitive function, but we also observed a consistent and significant worsening on other neurological domains with an increase in the number of individual MetS components. This phenomenon may be explained by the impairment of the frontal-subcortical network [39]. The ultrastructural breakdown of cerebral capillaries caused by cerebrovascular disease could disproportionately affect the frontal cortex and subcortical neural fibers that pass through the periventricular white matter, leading to selective cognitive, affective, executive, neuromotor, and mental dysfunction [4, 39].

## Conclusions

In conclusion, the results of the present study indicate that Chinese stroke patients with MetS have a greater risk of developing cognitive impairment, neuromotor dysfunction, and neuropsychological symptoms after acute cerebral ischemia. With the high prevalence of MetS throughout the world, even a modest association with cognitive impairment could have major public health implications. In light of the clinical relevance of dementia in public health, early identification and effective management to reduce metabolic risk factors may help to prevent or delay the onset of dementia syndromes and offer new avenues for disease course modification. There are some limitations in the current study. First, this was a single-center study, and thus, selection bias could not be avoided completely. Therefore, we have reduced the bias effect by adjusting for sociodemographic and clinical covariates. In addition, our investigation was cross-sectional and, consequently, cannot provide strong evidence for predicting changes in the cognitive function after stroke. Future follow-up of a larger cohort in our Cognitive Impairment Clinic Center (CIC) will provide more data for determining whether MetS at baseline and its combination with inflammation and oxidative stress biomarkers are associated with the increased risk of incident cognitive impairment and for elucidating the mechanisms underlying such associations.
